# Diuretic‐sensitive electroneutral Na^+^ movement and temperature effects on central axons

**DOI:** 10.1113/JP273963

**Published:** 2017-03-22

**Authors:** Meneka Kanagaratnam, Christopher Pendleton, Danilo Almeida Souza, Joseph Pettit, James Howells, Mark D. Baker

**Affiliations:** ^1^ Neuroscience and Trauma centre, Blizard InstituteQueen Mary University of London4 Newark StreetWhitechapelLondonE1 2ATUK; ^2^ Brain and Mind Research InstituteUniversity of Sydney94 Mallet StreetCamperdownNSW2050Australia

**Keywords:** excitability, nerve conduction, optic nerve, resting potential

## Abstract

**Key points:**

- Optic nerve axons get less excitable with warming.
- F‐fibre latency does not shorten at temperatures above 30°C.
- Action potential amplitude falls when the Na^+^‐pump is blocked, an effect speeded by warming.
- Diuretics reduce the rate of action potential fall in the presence of ouabain.
- Our data are consistent with electroneutral entry of Na^+^ occurring in axons and contributing to setting the resting potential.

**Abstract:**

Raising the temperature of optic nerve from room temperature to near physiological has effects on the threshold, refractoriness and superexcitability of the shortest latency (fast, F) nerve fibres, consistent with hyperpolarization. The temperature dependence of peak impulse latency was weakened at temperatures above 30°C suggesting a temperature‐sensitive process that slows impulse propagation. The amplitude of the supramaximal compound action potential gets larger on warming, whereas in the presence of bumetanide and amiloride (blockers of electroneutral Na^+^ movement), the action potential amplitude consistently falls. This suggests a warming‐induced hyperpolarization that is reduced by blocking electroneutral Na^+^ movement. In the presence of ouabain, the action potential collapses. This collapse is speeded by warming, and exposure to bumetanide and amiloride slows the temperature‐dependent amplitude decline, consistent with a warming‐induced increase in electroneutral Na^+^ entry. Blocking electroneutral Na^+^ movement is predicted to be useful in the treatment of temperature‐dependent symptoms under conditions with reduced safety factor (Uhthoff's phenomenon) and provide a route to neuroprotection.

## Key points

- Optic nerve axons get less excitable with warming.
- F‐fibre latency does not shorten at temperatures above 30°C.
- Action potential amplitude falls when the Na^+^‐pump is blocked, an effect speeded by warming.
- Diuretics reduce the rate of action potential fall in the presence of ouabain.
- Our data are consistent with electroneutral entry of Na^+^ occurring in axons and contributing to setting the resting potential.

**Abbreviations**
MS: multiple sclerosis
PNS: peripheral nervous system
QTRAC‐S: nerve stimulation and recording software

## Introduction

The circumstances in which Na^+^ enters axons is understood to be important for a number of reasons. These include the fact that the transmembrane Na^+^ gradient can run‐down where it is not continuously maintained (e.g. Baker *et al*. 1962), and the functional consequences of Na^+^ removal give rise to changes in the signalling properties of nerve (e.g. Rang & Ritchie, 1968; Bostock *et al*. 1991). Very importantly, energy is expended on removing Na^+^ ions (e.g. Friedrich *et al*. 1996), and this may become a critical issue where normal oxidative‐phosphorylation is impaired. Classical analysis has assumed that the important ways in which Na^+^ enters axons is always via ion channels, even when the nerve is at rest, and making this assumption has allowed the calculation of the contribution that the Na^+^,K^+^‐ATPase makes to the resting membrane potential (e.g. Fain, 1999). But if there are other, electroneutral routes for Na^+^ entry into nerve, then such calculations give an incomplete picture. Recently it has become clear that axonal degeneration is likely to be associated with energy deficit, and the importance of discovering new and effective pharmacological strategies for neuroprotection is a current imperative.

Damage to axons in neuroinflammatory, demyelinating disease is thought to be associated with a diminishing supply of ATP by a delayed cellular energy failure (Smith *et al*. 2001; Kapoor *et al*. 2003; reviewed by Waxman, 2003; Bechtold *et al*. 2005; Campbell & Mahad, 2012), and a subsequently raised intra‐axonal Na^+^. The acute effects of raising intra‐axonal Na^+^ through activity during a period of nitric oxide‐induced toxicity were demonstrated some years ago (Smith *et al*. 2001), and lead to axonal degeneration. It is widely appreciated that the intracellular Na^+^ ion concentration is important because transmembrane pumping of Na^+^ is a major drain on ATP production (e.g. Attwell & Laughlin, 2001). One consequence of inadequate control of the transmembrane Na^+^ gradient is the loss of control of intracellular [Ca^2+^] and ensuing cytotoxicity (e.g. Bei & Smith, 2012). Recently, the intracellular Na^+^ ion concentration in diseased human brain has been estimated by studying the distribution of ^23^Na using MRI (Petracca *et al*. 2016), revealing a significantly raised intracellular Na^+^ in the brains of people with multiple sclerosis (MS) compared with controls, consistent with impaired metabolism.

We have previously reported that properties of optic nerve axons are acutely temperature sensitive, with substantial changes in refractory period, afterpotentials and membrane potential occurring on warming. Our previous data suggested that raising the temperature from room temperature to near 37°C changes the membrane potential on average by −40 mV, and the effect is reversible with cooling (Coates *et al*. 2015). The biophysical properties of the axons suggest that the hyperpolarization is achieved without an increase in membrane conductance, and we have implicated the Na^+^‐pump coupled with an electroneutral Na^+^ movement into the axons in generating the effect. One reason that this may be clinically important is that electroneutral Na^+^ transport and exchange must necessarily increase with increasing temperature, providing a link between brain temperature and ATP requirement. There is also a likely tie‐up with symptom temperature dependence (Uhthoff's phenomenon) because hyperpolarization is expected to affect axonal excitability (Nelson & McDowell, 1959). Uhthoff's phenomenon was originally described as the worsening of symptoms in patients with optic neuritis associated with small increases in core body temperature (reviewed in Smith & Waxman, 2005). However, it is not only the visual system that is affected in this way. The worsening of symptoms and appearance of new symptoms with warming has been used historically as a diagnostic test for MS, and temperature‐dependent effects remain a common and major concern for people with MS, reducing quality of life (e.g. Malhotra & Goren, 1981; Leavitt *et al*. 2015). Temperature‐dependent effects remain inadequately treated with non‐steroidal anti‐inflammatory drugs and cooling suits. Another reason this may be clinically important is because the diuretic amiloride is already reported to reduce the rate at which brain mass is lost during progression in multiple sclerosis (Arun *et al*. 2013). Blocking electroneutral Na^+^ transport and exchange may therefore provide a new avenue for research into neuroprotection. The aim of the present study was to investigate the effects of changing temperature on the biophysical properties of central axons. By arranging similar changes in temperature in human peripheral axons, we compared the effects on recovery cycles, and note that the temperature sensitivity in the central nervous system and peripheral nervous system (PNS) axons appears to be different. In order to test further the hypothesis that warming hyperpolarizes the axonal membrane potential of optic nerve axons, we have studied refractoriness and action potential peak latency in the axons with the shortest latency responses (F‐fibres), and conclude that these properties change in a manner consistent with the hypothesis. A subsidiary aim was further to explore a potential mechanism of temperature sensitivity, the electroneutral leak of Na^+^ into the axons, and we approached this by blocking the Na^+^ pump, and following the collapse of the action potential at both near physiological and cool temperatures. Warming significantly increases the rate at which the action potential declines, consistent with the proposed mechanism, and drugs blocking electroneutral Na^+^ ion transport and exchange slow the decline at near physiological temperatures.

## Methods

### Optic nerve isolation

All animal work carried out conformed to UK Home Office legislation (Animals (Scientific Procedures) Act 1986). Optic nerves isolated from male Wistar rats (∼320 g) and C57bl/6 mice (> 10 weeks, either sex), obtained from Charles River Laboratories were used in this study. Animals were housed at a designated establishment and had access to food and water *ad libitum*. They were killed in accordance with UK Home Office guidelines (Schedule 1) by a rising concentration of CO_2_, followed by cervical dislocation. The investigators understand the ethical principles under which *The Journal of Physiology* operates and this work complies with the animal ethics checklist. The optic nerves and eyeballs, were quickly released from their sockets, placed in oxygenated buffer and cleaned of adherent tissue, in order to facilitate mounting in an *ex vivo* nerve bath.

Essentially, the methods have been fully detailed elsewhere (Coates *et al*. 2015). Two designs of rodent optic nerve bath were developed that allowed recording from mouse and rat nerve, which have substantial size differences. The rat optic nerve was large enough to be pulled across a hollow barrier between two chambers containing buffer, with the barrier subsequently filled with petroleum jelly. The eyeball remained in the left‐hand chamber. In the right, the stimulating electrode was placed and solution perfused, allowing the application of drugs and changes in temperature to almost all of the nerve. The bath for the mouse nerve comprised two chambers, separated by a narrow plastic wall. In the larger of the chambers, almost all of the optic nerve was bathed in buffer solution, while in the smaller chamber, the eyeball was completely surrounded by petroleum jelly, in effect making the eyeball the left‐hand chamber in the recording arrangement.

More than one fibre group contributes to responses of rat optic nerve and in order reliably to record changes in latency, we chose to study the fast F‐fibre group in functional isolation, with the smallest stimulus currents, and the briefest corresponding stimulus artefact. These are likely to be the largest axons in the optic nerve, expected to be most similar to peripheral axons, and hence most easily compared. The expectation was they will be most affected by any slowing in conduction velocity elicited by temperature changes.

### Solutions and drugs

Recording solutions contained (in mm): NaCl 140, hemisodium Hepes 10, CaCl_2_ 2.1, MgCl_2_ 2.12, KCl 2.5 and glucose 10, adjusted to pH 7.2–3 using HCl. The applied solutions were oxygenated after passing through a heating jacket (HPT‐2, ALA Scientific, Farmingdale, NY, USA). The temperature in the bath, was measured using a local thermistor close to the nerve, and the value of the temperature kept at the desired level by negative feedback. Temperature was continuously recorded using an analogue input channel on QTRAC‐S nerve stimulation and recording software (developed by Hugh Bostock, Institute of Neurology; Digitimer, Welwyn Garden City, UK). Flow rate was close to 2 ml min^−1^.

Bumetanide, amiloride and ouabain were obtained from Tocris (R&D systems, Bristol, UK), made up as stocks in dimethyl sulphoxide, stored frozen at −20°C, and diluted when required in buffer solution. The concentration of vehicle was never as high as 0.1%. All salts and buffers required for making solutions were obtained from Sigma‐Aldrich (Dorset, UK).

### Stimulation and recording

In the *ex vivo* preparation stimulation and recording was carried out as detailed in Coates *et al*. 2015, using the stimulation and recording program QTRAC‐S, with the exception that for threshold‐tracking we usually used either < 10% or exactly 5% of the supramaximal peak action potential amplitude as measured at room temperature as the target amplitude to be maintained, rather than a 50% response. This means that the axons making up the tracked response were overwhelmingly of the F‐fibre type. Periodically the amplitudes were checked for consistency, and in some experiments reported here, the supramaximal action potential was continuously recorded. While there were small changes in action potential amplitude during changes in temperature, in fact, as previously reported, the amplitude in normal buffer solution is quite stable (Fig. 3), we believe, in large part, because of the effect of Na^+^ channel inactivation becoming progressively relatively more rapid at warmer temperatures, and hiding the latent increase in amplitude expected by an effect on resting membrane potential.

The fastest rate of stimulation for both threshold‐tracking and following compound action potential amplitude was 1 Hz. Regarding the supramaximal response, at the top of the recruitment curve the relationship between changing stimulus current and the response amplitude is flat, and being supramaximal in stimulus amplitude at the start of recording, near room temperature, it is unlikely we would see much change in amplitude with temperature change even if the stimulus was not always supramaximal to the same extent. Recordings were stable over tens of minutes, and we do not believe that slow inactivation of Na^+^ channels was building up at the protocol repeat frequency utilized, as action potential latency did not change.

**Figure 1:**
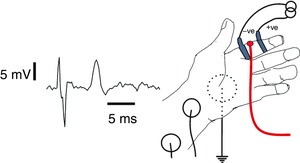
Experimental arrangement for threshold‐tracking orthodromic volleys in D2 sensory axons Left, example median nerve sensory tracked response is shown, preceded by a stimulus artefact. Right, the stimulation and recording arrangement. Constant‐current stimulation in the finger was applied via ring electrodes. Recordings were made proximal to carpal tunnel above median nerve. Temperature was monitored at D2 skin surface close to the stimulating cathode (thermistor indicated as a disk). [Color figure can be viewed at wileyonlinelibrary.com]

We arranged for human normal peripheral nerve to undergo large swings in temperature by studying the properties of afferents in D2, first finger, recording the evoked sensory nerve action potential at the wrist (Fig. 1). Details of methods and purpose built pre‐amplifier can be found in Howells *et al*. 2013. D2 offers advantages, in that the afferents innervating the skin are close to the surface, and therefore close to the applied temperature probe. Secondly, the finger normally undergoes large changes in temperature, and finally finger temperature is easy to alter. D2 afferents were stimulated using ring electrodes. Stimulus currents were applied using a computer‐controlled constant‐current device (DS5, Digitimer) and the evoked sensory volley in the median nerve recorded at the wrist. Threshold was tracked at 40% of the maximal volley amplitude. Changes in finger temperature could be produced without difficulty using warmed microwavable wheat‐filled bags, and freezer‐cooled cold‐pillows, applied where necessary. In order to keep finger temperature stable at a temperature higher than ambient, the hand was covered with a flannel towel. The control software was QTRAC‐S, which aimed to maintain a constant response at the wrist, while temperature changes were applied to the finger, so that threshold was measured in terms of the stimulus current necessary to evoke the target response amplitude. Informed and consenting adults were recruited to undergo recording of current threshold with changing finger temperature, and also the recording of recovery cycles at room temperature and at 35–37°C. Ethical permission was obtained from the University of Sydney, and the experiments conform to the *Declaration of Helsinki*.

### Data analysis

Wherever possible data are plotted as means ± SEM. Statistical comparisons were made using SPSS Statistics version 22 (IBM, Armonk, NY, USA) or Microsoft Excel 2007. Data sets were compared using paired, unpaired and one‐sided Student's *t* test, when appropriate, and in the event of multiple comparisons, the Holm–Bonferroni correction technique was used.

## Results

**Figure 2:**
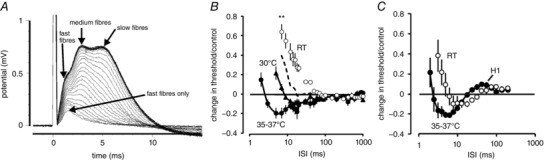
F‐fibres are the population of axons in the optic nerve appearing with the shortest latency; their response to changing temperature differs from that of finger afferents *A*, supramaximal response at room temperature (RT) can be seen to comprise three components, F, M and S. Progressively reducing the stimulus current in QTRAC to allow tracking at 5% reduces the response amplitude and shifts the peak latency leftward, until only the F‐fibre response remains. *B*, raising the temperature gives rise to a dramatic shortening of refractory period, and increase in superexcitability. F‐fibres were tracked at < 10% of stimulus current necessary to elicit a supramaximal response. Inter‐stimulus interval (ISI) stepped gradually from 2 ms to 1 s during the protocol. By raising the nerve temperature from RT to 35–37°C, a near 100 ms refractory period was reduced to near 2 ms, in an incremental manner (dashed line, single nerve maintained at 27°C). At an ISI of 7 ms, *P* = 0.0019 comparing only paired data at RT and 35–37°C (*n* = 3), indicated as ^**^. Total *n* was 6, 4 and 3 for RT, 30°C and 35–37°C, respectively. *C*, human finger afferents show a much smaller change in refractory period duration over the same temperature range (*n* = 3, *n* = 4, 35–37°C and RT, respectively; comparing paired‐data differences are non‐significant), but a similar refractory period duration at warm temperatures as the F‐fibres, followed by a noticeable sub‐excitability, H1, not apparent in the optic nerve responses.

Rodent optic nerve is understood to comprise three functional groups of myelinated nerve fibres, which we have previously referred to as F, M and S (Coates *et al*. 2015), but which have been reported many times by several groups of authors (e.g. Devaux & Gow, 2008). The F (fast), M (medium) and S (slow) components contribute to early, middle and late parts of an evoked supramaximal action potential. When stimulating with a brief (200 μs) current stimulus, a large stimulus current evokes all three components. When using a smaller current and generating a response only 50% of the supramaximal amplitude, the response is generated by the F and M fibres, and a current capable of recruiting less than 10% evokes an action potential generated by F‐fibres. The F‐fibres appear to be the axons with the fastest conduction velocities and shortest induction times. They have the lowest threshold to a brief stimulus, presumably with the largest diameters and shortest rheobase (Fig. 2 *A*).

### F‐fibre recovery cycles

Although a number of factors might influence the amount of current required to generate an appropriate response for threshold‐tracking, recovery cycles are thought of as a relatively robust measure of axonal function, and independent of the absolute current, because when generating recovery cycles the current required following a conditioning stimulus and response is expressed as a proportion of the control current, measured in an adjacent channel in QTRAC. So, recovery cycles are independent of the absolute value of stimulus current applied, as the increase or decrease in threshold is expressed relative to a simultaneously measured control threshold value.

One difference between the reported optic nerve recovery cycle at physiological temperatures (Coates *et al*. 2015) and those reported for peripheral axons is that the magnitude of superexcitability following an action potential was smaller than for peripheral nerve (cf. Tomlinson *et al*. 2010). Previous threshold‐tracking experiments in optic nerve were performed by maintaining the response at 50% of maximal. However, we can now report that this does not seem to be the case for the F‐fibres in rat optic nerve axons, as recording recovery cycles for axons contributing to the first 10% of axons recruited produced similar responses to those seen in the periphery near physiological temperatures (Fig. 2 *B* and *C*). Additionally, these F‐fibre responses also showed the same remarkable changes in refractory period with changing temperature that we had seen before (Coates *et al*. 2015) and previously suggested may be caused by a temperature‐sensitive membrane potential. So the only obvious difference between tracking by recruiting only F fibres or both F and M is that the superexcitable period at body temperature is larger, conceivably because the F‐fibres, and their myelin sheaths, have a microanatomy more closely similar to peripheral myelinated axons.

Increases in finger temperature consistently gave rise to simultaneous, small increases in threshold, indicated by a rise in necessary stimulus current (13.1 ± 3.4%, *n* = 5, mean ± SEM with an average temperature change away from ambient of +8.4°C). Although this is statistically significant (*P *< 0.02; one‐sided *t* test) and an increase in threshold is consistent with a hyperpolarization of the membrane potential, it is a considerably smaller effect than that found for optic nerve fibres. Using a rule‐of‐thumb conversion to the responsible change in membrane potential (Kiernan & Bostock, 2000), this indicates large peripheral afferents probably increase their membrane potential by only −2 to −3 mV for a 10°C rise in temperature, entirely in line with the findings of Howells *et al*. (2013). Comparing the recovery cycles in F‐fibres with those recorded in the human finger afferents reveals two notable differences in behaviour. Although subjecting finger afferents to changes in temperature similar to those achieved in the *ex vivo* nerve bath, the refractory period in the cold finger is not significantly longer than that following warming to near physiological temperature (Fig. 2 *C*). Furthermore the finger afferents display the clear late‐subexcitability attributable to the activation of kinetically slow K^+^ channels (H1, e.g. Tomlinson *et al*. 2010), and this is not seen in the F‐fibre responses at any temperature, suggesting a difference in the function and distribution of K^+^ channels between the peripheral and central axons.

### The action potential amplitude and impact on threshold

**Figure 3:**
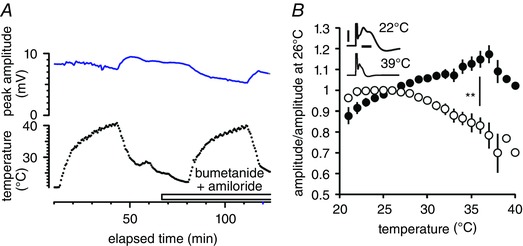
The combination of amiloride and bumetanide alters the way in which the optic nerve supramaximal action potential responds to warming *A*, example raw data set showing the effect of changing temperature (lower panel) on peak action potential amplitude (upper panel). Drugs introduced during period indicated with open bar, with time allowed for equilibration. Raising temperature has almost no effect on compound action potential amplitude without drugs, excepting a slight fall at the highest temperatures. In the presence of amiloride and bumetanide, the amplitude falls on warming. *B*, paired amplitude data from 5 nerves without (filled circles) and with 10 μm amiloride and 5 μm bumetanide (open circles). Amplitude is normalized with respect to the value measured at 26°C in each condition. On average, the amplitude of the action potential undergoes an increase as the temperature is raised from RT, but in the subsequent presence of electroneutral Na^+^ transport blockers, amplitude consistently falls (*P* = 0.0075, paired *t* test at 36°C, ^**^). Inset, example action potentials recorded at room and near physiological temperatures, in the presence of amiloride and bumetanide, show the temperature‐dependent fall in amplitude. Scale bars 5 mV and 2 ms. [Color figure can be viewed at wileyonlinelibrary.com]

The threshold‐tracking technique seeks to maintain a constant response from a group of nerve fibres, and one factor that can influence the amount of current applied is how easy it is to recruit the axons, though this is not the only factor. If properties of the individual (unresolved) impulses alter, for example the impulse amplitude falls with raising temperature, then the threshold‐tracking program (QTRAC‐S) will pass more current, in order to recruit more axons and maintain the response. Because changes in supramaximal action potential amplitude were measured during solution temperature changes, it became clear that the interpretation of absolute threshold changes in optic nerve was not straightforward. Acknowledging this fact, we found that changes in action potential amplitude were, if anything, in the direction that would tend to make the measured threshold fall (Fig. 3), although comparing the limits of the temperature range studied, the change in amplitude was small or non‐existent. We reported in Coates *et al*. (2015) that there was no change in amplitude or a slight reduction and any discrepancy with the present findings we attribute to variations in individual nerve recordings, although the findings are similar. However, for action potentials recorded in the presence of bumetanide (5 μm) and amiloride (10 μm), blockers of electroneutral Na^+^ movement, a consistent fall in action potential amplitude of near 30% was found (Fig. 3), highly reminiscent of the findings of Hodgkin & Katz (1949) in squid giant axons, where the impulse amplitude fell with warming and where there is known to be no change in resting membrane potential. The fall in action potential amplitude in the squid axon experiment is explained by the greater sensitivity of Na^+^ channel inactivation than activation to warming, so the action potential is truncated and the peak falls in a temperature‐sensitive manner (Hodgkin & Katz, 1949). Regarding our present findings, we suggest that there are two implications. Firstly, the presence of the drugs must reduce any change in membrane potential associated with warming, and this is why the action potential amplitude falls. Secondly, trying to derive real temperature‐dependent changes in threshold in the presence of the drugs must be frustrated by the fall in action potential amplitude, tending to artefactually drive the threshold measurement upward on warming. Therefore we did not attempt to draw conclusions about axon excitability in the presence of bumetanide and amiloride.

### F‐fibre threshold tracking

**Figure 4:**
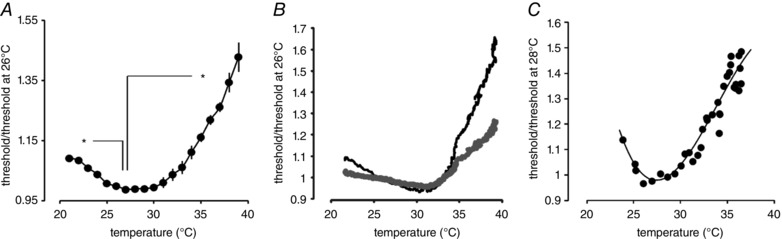
Current threshold changes with temperature *A*, raising temperature from RT to near to physiological results in threshold changes, including an initial decrease, and subsequently an increase. From 22 to 27°C, threshold falls by 8.5% (*P* = 0.004, *n* = 5, paired *t* test); from 27 to 38°C, the threshold increases by 36% (*P* < 0.001, *n* = 10, paired *t* test). Comparisons remain significant following Holm–Bonferroni correction (^*^). Data normalized to threshold value at 26°C. *B*, example recording where threshold tracking at 5% (black trace) took place with simultaneous measurement of peak supramaximal action potential amplitude. A part of the excitability change is explained by change in the amplitude of the action potential, with an inverse relation between action potential amplitude and the current required to maintain tracked response. Predicted changes in threshold are plotted based on the changes in supramaximal compound action potential amplitude, and assuming an inverse relation (grey trace); data normalized to threshold value at 26°C. *C*, similar threshold changes occur in mouse nerve, tracked at 50% of maximal amplitude. Smooth curve is a best‐fit polynomial and of no theoretical significance.

For a change in membrane potential of −40 mV (suggested by the findings of Coates *et al*. 2015), one might expect to see changes in absolute threshold, and we can report there are increases in threshold associated with warming in both rat and mouse axons. When tracking at 5% of maximal amplitude in the rat nerve there were consistent changes in threshold, where overall the threshold at near physiological temperatures is significantly higher than at room temperature (Fig. 4 *A*). Very similar changes in threshold have also been seen in mouse optic nerve (Fig. 4 *C*). Why the threshold changes are not larger is discussed in more detail below, but the magnitude of the effect appears similar to changes in threshold seen during 15 min of ischaemia for human forearm nerve (Kiernan & Bostock, 2000). The simplest hypothesis relating to the functional effects of a membrane potential hyperpolarization would be that axons should become harder to stimulate, and our data suggest this is indeed the case. In essence then, this hypothesis is confirmed. This finding, in itself, does not prove a warming‐induced steady‐state hyperpolarization is operating in optic nerve axons, because an increase in Na^+^ channel inactivation rate over and above a change in activation rate brought about by warming (Hodgkin & Katz, 1949) might cause an increase the threshold. However, we suggest that while the presence of a warming‐induced hyperpolarization will require independent proof of another kind, a hyperpolarization is also consistent with the type of temperature‐dependent changes in recovery cycle we have described (Fig. 2 *B*).

### F‐fibre impulse latency

**Figure 5:**
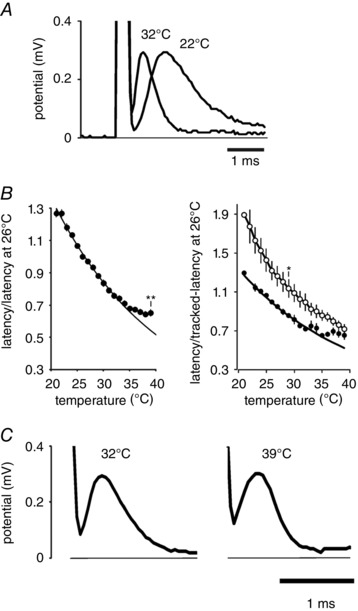
Peak latency of the F‐fibre component falls with temperature but latency change slows and stops when the nerve is warmed above 30°C *A*, example 5% tracked responses at 22 and 32°C show the dramatic increase in rate of action potential up‐ and down‐swing, resulting in a fall in peak latency. *B*, left, latency data between RT and 30°C are well fitted by eqn (1) (1.237 and 1.631, *L* at 22°C and *Q*_10_, respectively), but there is an apparent discrepancy at warmer temperatures with the latency values longer than predicted (at 39°C, mean latency differs from predicted value *P* = 0.007, one‐sided *t* test, *n* = 7, ^**^). Right, with warming peak latency of tracked response approaches that of the supramaximal response. Tracked responses and supramaximal peak latencies (in the same nerves), filled and open circles, respectively, *n* = 4. At 29°C, values were significantly different (*P* = 0.042, paired *t* test, *n* = 4, ^*^) but at temperatures above 30°C, the tracked response latency approaches the supramaximal peak latency, which should always be longer because of the recruitment of slower components. Smooth curve through tracked‐data given by eqn (1), with 1.21 and 1.631, *L* at 22°C and *Q*_10_, respectively. *C*, there was no fall in peak latency between 32 and 39°C. Peak latency minimum is not caused by an inability to resolve the action potential, occurs in spite of an increase in the speed of down‐swing, and does not appear to be caused by the recruitment of the M component.

As the absolute threshold measurement with changing temperature is likely to be affected to some degree by the amplitudes of the individual action potentials, we have also tried an alternative approach to understanding the functional effects of warming optic nerve axons, by measuring the peak latency of F‐fibre volleys. Latency provides a physiologically meaningful measurement that would be expected to be less dependent on changes in action potential amplitude. Furthermore, latency measures might be expected to be impacted by changes in conduction velocity and also induction time, both of which will be affected by alterations in the membrane potential of axons. One issue quickly dealt with is that most of the change in peak latency occurring on warming in the nerve bath we used is caused by a change in the shape of the action potential on warming, rather than another variable (Fig. 5 *A*), a factor also described for recordings in human peripheral nerve (Bolton *et al*. 1981).

We could approximately predict changes in F‐fibre peak latency using a simple equation accounting for changes in action potential kinetics and defining a *Q*_10_:

(1) l=L/Q10(Δ Temp /10)

Where *I* is the peak latency at any temperature (ms), *L* is the peak latency value at the reference temperature (ms), *Q*_10_ is the temperature coefficient, and ΔTemp is the difference in temperature from the reference (°C).

For the tracked responses, while eqn (1) appeared acceptably to predict latency changes on increasing temperature up to 30°C, the relationship broke down with warming to higher temperatures (Fig. 5 *B* and *C*). Putting this finding in context, evidence from studies in peripheral nerve in man has suggested that the relationship between conduction velocity and temperature becomes flatter at temperatures approaching normal physiological values (Todnem *et al*. 1989), so that in the PNS increasing temperature does not always make the impulse arrive more quickly, and why this occurs, at least to our knowledge, is unknown. One possibility is that hyperpolarization occurs in peripheral nerve also.

### Impulses slow down in warm axons

Plotting the peak latency of the supramaximal response and the 5% tracked response in the same nerve, over a range of temperatures, indicated a possible slowing of the tracked response as the temperature was increased (Fig. 5 *B*). The response to a large supramaximal stimulus generated an action potential with an expected peak latency longer than that of the F‐fibre component (cf. Fig. 2), because M‐fibres contribute to the overall waveform. At cool temperatures, the F‐fibre component was clearly at a shorter latency than the supramaximal action potential in the same nerve, but we found that this difference was mostly eliminated on warming, suggesting that the axons excited by a near threshold current had a longer combined induction and conduction time when the nerve was warm (Fig. 5 *B* and *C*), and this slowed down the arrival of the peak. This slowing of the peak was found in spite of a more rapid action potential downswing that can be seen in Fig. 5 *C*, reflecting the effect of temperature on ion channel kinetics. In one nerve there was a likely M‐fibre component added to the 5% response when the nerve was warm, which gave rise to a broad action potential peak, and these data were removed from the analysis. That the result of a near threshold excitation should be much more sensitive than the response to a well supramaximal stimulus current may be explained by the absolute size of the conduction velocity change being larger in the faster conducting fibres.

### Na^+^‐pump inhibition causes maximal action potentials to collapse

**Figure 6:**
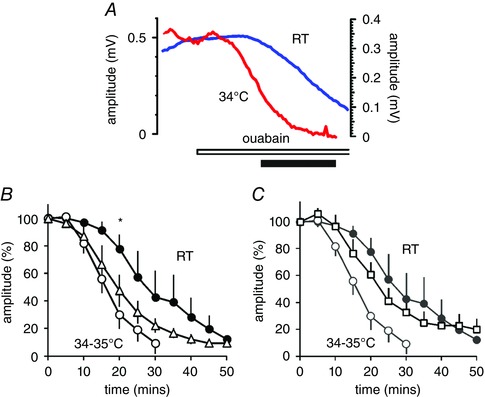
Ouabain causes the supramaximal action potential in mouse nerve to collapse Raising temperature speeds the decline, and the addition of a combination of bumetanide and amiloride reverses the effect of raising the temperature. *A*, example plots of raw peak supramaximal action potential amplitude, before and after the application of 40 μm ouabain, at RT and 34°C. Left‐ and right‐hand *y*‐axes for RT and 34°C, respectively. Both plots show the first 50 min of recording, and the ouabain is applied after 9 min in both cases. Filled time‐bar indicates 20 min. *B*, once the decline is started, raising the temperature significantly speeds the decline (filled circles and open circles, room temperature and at 34–35°C, respectively, *n* = 4, 5, at 20 min *P* = 0.014, unpaired *t* test). At 34–35°C, the addition of bumetanide (5 μm; open triangles, *n* = 6) and subsequently, *C*, bumetanide and amiloride (5 μm plus 10 μm; open squares, *n* = 4) reverses the effect of raising temperature. Data already presented in *B* shown in grey in *C*. [Color figure can be viewed at wileyonlinelibrary.com]

We needed an alternative approach to provide another line of evidence that Na^+^ fluxes into axons changed with temperature, and we chose to examine the way in which a supramaximal action potential collapses when the Na^+^ extrusion mechanism is compromised. Exposure of mouse optic nerve to ouabain (40 μm) gives rise to a gradual loss of maximal action potential amplitude over tens of minutes. As this concentration it is expected that ouabain will partially block the Na^+^‐pump, known to be present in optic nerve (Gordon *et al*. 1990). The likely explanation for the phenomenon is that the electrochemical gradient for Na^+^ across the axon membrane runs down, despite ATP production in the nerve being unaffected. Comparing the decline at steady‐state room temperature to steady 34–35°C revealed that the warming speeded the decline by a factor of close to 2 (*P* = 0.014, unpaired *t* test) (Fig. 6 *A* and *B*), suggesting that raising the temperature must increase the rate of entry of Na^+^ into axons. This result is unlikely to be explained if Na^+^ channels provide the main route of entry (as Na^+^ channels would be predicted to be open for less time at higher temperatures, through the effect of temperature on channel gating), but it is consistent with the idea that most Na^+^ influx is mediated by ion transporters and exchangers, whose rate of operation increases with increasing temperature.

Following application of bumetanide (5 μm) plus amiloride (10 μm), the rate of action potential decline at a steady 34–35°C became not significantly different from the decline at room temperature (Fig. 6 *C*), suggesting that electroneutral transport and ion exchange are responsible for some of the Na^+^ entry into optic nerve axons, and also supporting the position that it is raised Na^+^ influx that causes the collapse of the action potential. These findings indicate that blockers of Na^+^ transport can be used to reverse the effects of raising temperature on central white matter, and given that uncontrolled intra‐axonal Na^+^ can be toxic, such drugs may offer neuroprotection.

## Discussion

Our data indicate that raising temperature from room to near physiological increases the threshold of F‐fibres, and while there may be questions about how accurately the threshold change may be ascertained in a multi‐fibre preparation, the finding is consistent with the proposal that central axons hyperpolarize when they warm. In contrast, the effect of a similar increase in temperature in human finger afferents indicates that if hyperpolarization occurs in PNS axons, it is substantially smaller. A previous analysis of optic nerve data suggested that the hyperpolarization was caused by a temperature‐dependent electroneutral flux of Na^+^ into axons (Coates *et al*. 2015), coupled with electrogenic expulsion of the ion by the Na^+^‐pump, and complementing this interpretation, we have further found that electroneutral Na^+^ transport blockers caused the action potential to fall in amplitude as the nerves were warmed, suggesting that normally Na^+^ influx causes a hyperpolarization and an increase in the amplitude of action potential up‐swing. Finally we have provided evidence for a temperature‐dependent process causing the latency of the F‐fibre volley to be prolonged; in fact at temperatures near physiological, the action potential latency for the tracked response had essentially stopped shortening as the temperature increased above 30°C.

### Difference between PNS sensory and optic nerve axon response to warming

Temperature sensitivity in human peripheral axons has been reported before (e.g. Burke *et al*. 1999; Howells *et al*. 2013), and the available data, including those presented here, point to the temperature sensitivity being rather modest and associate warming with only a small increase in threshold. This suggests only a few single millivolt change in membrane potential when the ambient temperature changes over 10°C. Our estimates from demarcation potential recordings in rodent optic nerve (Coates *et al*. 2015) indicate that the central axons may change their membrane potential something like 5–10 times more for the same increase in temperature. This difference may partly reflect the operating environments for peripheral and central axons, where it is beneficial that sensory afferents in the periphery should retain stable signalling while subjected to a range of temperatures. In contrast, central axons are normally subject to only small changes in core body temperature, and under these circumstances another factor, such as the control of intracellular Cl^−^ or pH in the neuron, and the axon, may be the more important consideration, for example in the fine tuning of inhibitory neurotransmission.

### Size of the observed threshold change in optic nerve

If membrane potential alters by tens of millivolts on warming from room temperature to near physiological values, why are the threshold changes rather small? On average, comparing threshold at room and near physiological temperature, there is an increase of less than 50% for the F‐fibres. We have found that individual nerves reveal some variability, with F‐fibre measurements showing a doubling of threshold. The first and most obvious explanation is that it is normal for action potential amplitude to increase a little with warming across the temperature range employed (Fig. 3), and this means that the tracking program will need to recruit fewer axons with warming and thus any increase in threshold will be underestimated by our technique. However, it is already estimated for normal human peripheral nerve that a 1 mV depolarization should reduce threshold by about 8% (Kiernan & Bostock, 2000), making the assumption that a just‐threshold stimulus at rest changes the nodal potential by about 10–15 mV. If this prediction were applicable in our experiments, changing temperature from room to near physiological should apparently change nodal membrane potential by about −6 mV, rather than −40 mV for the average value previously estimated from demarcation potential recordings in whole optic nerve. For comparison, temperature‐dependent changes in membrane potential in cortical pyramidal cells of a size similar to −40 mV have already been published (Volgushev *et al*. 2000). The disparity from the Kiernan and Bostock rule‐of‐thumb may be explained because the relationship between the threshold and membrane potential is non‐linear with depolarization, partly because of the activation of *G*_Ks_ (KCNQ, K_V_7.2/7.3; Schwarz *et al*. 2006, Battefeld *et al*. 2014; cf. Kiernan & Bostock, 2000, their Fig. 3 *A* and *C*), but also increasing Na^+^ channel inactivation (Bostock & Grafe (1985), their Fig 11), and the value derived by Kiernan and Bostock is from brief applications of current that do not change the activation state of *G*_Ks_. So, importantly, there must be functional differences between the effect of brief changes in membrane potential (affecting primarily the nodes) and the effect of long‐lasting changes in membrane potential. Exploring this theme, the closest approximation we have found in the literature to the effects of changing the temperature between near physiological and room in optic nerve is the effect of the application of 15 min of ischaemia to peripheral nerve (Kiernan & Bostock, 2000, their Fig. 2, responses I2). The ischaemic human nerve in Kiernan and Bostock's (2000) paper has a threshold measured close to 2 mA, whereas that in the normal nerve was near 3 mA, proportionally very similar to the threshold changes we are reporting here for F‐fibres.

Earlier studies on squid axon (Hodgkin & Katz, 1949; Fitzhugh, 1966; Guttman, 1966) show that raising the temperature can make axons more excitable by increasing the rate of Na^+^ channel activation, so the channels respond more quickly to applied currents; however, our attempts at simulation have suggested that any hyperpolarization with rising temperature obviates this effect. Resting *G*_Ks_ activation will be removed as the axons hyperpolarize (decreasing the resting conductance of the node with hyperpolarization) (Dubois, 1981; Baker *et al*. 1987), as will resting Na^+^ channel inactivation (Schwarz *et al*. 1995; Chen *et al*. 2008). These changing parameters represent mechanisms that could, at least in principle, contribute to a real increase in excitability on warming. If one accepts that membrane potential increases with warming, then occurring at the same time, action potential amplitudes can be made bigger through an increase in resting membrane potential (Coates *et al*. 2015). Alternatively, *E*_Na_ may be shifted positive with an increased rate of Na^+^‐pumping, potentially depleting the intra‐axonal Na^+^ concentration. Larger action potentials subsequently will cause a reduction in necessary stimulus current because fewer axons will need to be recruited to the response to maintain the required amplitude. In line with these considerations, normally, when warming from room temperature, there is a fall in threshold, and our analysis suggests that a major fraction of that can be attributed to individual action potentials getting larger, as evidenced by the change in supramaximal action potential amplitude (Fig. 3). It is a falling threshold, then, that dominates in the range between room temperature and 30°C, and the predicted real increase in threshold occurs at higher temperatures. We suggest that the changes in threshold reported here, though highly stereotypical, result from the interplay of a number of factors. Tracking single units in optic nerve has not yet been achieved, but such a technique may eliminate problems with the amplitude of individual action potentials changing over the applied temperature range and so help resolve the effects of temperature more clearly.

Another question is, what might limit the extent of any temperature‐dependent hyperpolarization and so limit any temperature‐dependent loss of excitability? We propose that the activation of *I*_h_ (HCN channels), with hyperpolarization is likely to do this because there is evidence the HCN channels are expressed in central axons (Eng *et al*. 1990; Garthwaite *et al*. 2006), and that they can counter Na^+^‐pump‐mediated hyperpolarization (Baker *et al*. 1987).

### Action on tracked‐response latency by warming optic nerve above 30°C

We turned our attention to the effects of warming on action potential latency, and our findings indicate that warming affects peak latency most through changing the action potential shape, reducing peak latency as the nerve gets warmer. More subtle changes in this latency measure, almost certainly related to conduction velocity and induction time, are a surrogate for changes in excitability and must also be affected by any biophysical properties of the axons impacting excitability. We suggest that the latency changes have advantages over measuring threshold because they are not dependent upon action potential amplitude *per se*. At temperatures higher than 30°C, the peak amplitude of the tracked F‐component becomes delayed with respect to the peak maximal action potential recorded in the same nerve, and this suggests that some process impacts conduction velocity and/or induction time, which might be interpreted as indirect evidence for axon hyperpolarization. Additionally, the effect of altered conduction velocity is easiest to see in our preparation for the fastest conducting F‐fibres, because that is where the absolute change in conduction velocity is going to be largest.

### Bumetanide and amiloride protect the action potential

The maximal peak action potential declines in the presence of ouabain, consistent with loss of control of the intra‐axonal Na^+^ ion concentration. This situation is exacerbated by warming, but the application of Na^+^ transport blockers slows the decline, in a manner similar to that expected for cooling. Our data suggest that two ways in which Na^+^ enters axons electroneutrally are the anion–cation co‐transporter (NKCC1), and the Na^+^/H^+^exchanger (NHE; Coates *et al*. 2015), but it further suggests that other ways for Na^+^ to enter are likely, because even in the presence of high concentrations of these two drugs there is still a decline in the action potential amplitude. For transmembrane Na^+^ leakage to lead to hyperpolarization there must be more than twice as much Na^+^ entering electroneutrally at rest than through ion channels because of the Na^+^/K^+^‐ATPase ion exchange stoichiometry. Electroneutral Na^+^ transport blockers may therefore warrant more study as potential neuroprotective agents. As evidence already exists for a neuroprotective effect of phenytoin in optic neuritis (Raftopoulos *et al*. 2016), one possibility is that simultaneously targeting more than one Na^+^ entry route (e.g. channels and transporters) might enhance the protection offered to axons.

## Additional information

### Competing interests

The authors declare there are no competing interests.

### Author contributions

M.K. performed experiments undertook analysis and made figures; C.P. performed experiments and undertook analysis and made figures; D.A.S. performed experiments undertook analysis and made figures; J.P. performed experiments; J.H. performed experiments; and M.D.B. devised experiments, performed experiments, undertook analysis and wrote the manuscript. All authors have approved the final version of the manuscript and agree to be accountable for all aspects of the work. All persons designated as authors qualify for authorship, and all those who qualify for authorship are listed.

### Funding

We acknowledge the support of the Neuroscience intercalated degree program, QMUL (M.K., C.P.), Students Without Borders program, QMUL (D.A.S.) and the MS society UK (innovation award 16, M.D.B. and J.P.).
